# Reemergence of *Brucella abortus*, Israel, 2021

**DOI:** 10.3201/eid3104.241003

**Published:** 2025-04

**Authors:** Svetlana Bardenstein, Daniel Grupel, Boris Even-Tov, Yair Motro, Jacob Moran-Gilad

**Affiliations:** National Brucella Reference Laboratory, Kimron Veterinary Institute, Ministry of Agriculture and Food Security, Beit Dagan, Israel (S. Bardenstein); Hadassah-Hebrew University Medical Center, Jerusalem, Israel (D. Grupel, J. Moran-Gilad); Galil-Golan Regional Office, Veterinary Services, Ministry of Agriculture and Food Security, Rosh Pina, Israel (B. Even-Tov); School of Public Health, Faculty of Health Sciences, Ben Gurion University of the Negev, Beer Sheva, Israel (Y. Motro, J. Moran-Gilad).

**Keywords:** brucellosis, bacteria, zoonoses, outbreak, bovine, One Health, genomic, surveillance, Israel

## Abstract

After nearly 4 decades, *Brucella abortus* has reemerged in Israel, triggering an outbreak across 2 dairy farms (82/137 cows affected), as well as cases in dogs and 1 human case. Despite thorough epidemiologic and genomic investigation, the outbreak source remains unidentified. Such reemergence poses One Health challenges and necessitates ongoing surveillance.

Brucellosis is a zoonotic bacterial infection that has a substantial effect on human and animal health. Most cases involve *Brucella melitensis*, for which sheep and goats are the natural reservoirs, or, to a lesser extent, *B. abortus,* for which cattle are the reservoir. *B. melitensis* is endemic in Israel, causing sporadic human cases and outbreaks (*1*,*2*), and has recently been implicated in the spillover of infection to dairy cow herds and secondary human infections (*3*). In contrast, *B. abortus* has been absent in Israel since 1985, after cattle vaccination and control measures were put in place (*4*); cattle vaccination was subsequently discontinued in 2018. Only 3 sporadic human *B. abortus* cases have been identified in recent years, all imported from known endemic countries. We report on the investigation of *B. abortus* reemergence in Israel after 40 years of elimination and discuss its implications.

## The Study

In late 2021, *B. abortus* was detected in a culture of third-trimester abortion material obtained from a dairy cow in an agricultural community (Moshav) in northern Israel. This community hosts several small cattle farms (dairy farming and feedlots). The investigation protocol triggered by *Brucella* detection in dairy farms includes serologic screening of all cattle at the farm, followed by confirmatory selective milk culture from seropositive cows. Characterization of growing isolates is performed at the National Reference Laboratory for brucellosis (NRL) (Kimron Veterinary Institute, Beit Dagan, Israel).

Investigation at the dairy farm (farm 1) identified 40 (66.7%) of 60 cows that were seropositive for *Brucella*, and 7 abortion cases were detected. Milk cultures were obtained and confirmed positive for *B. abortus* from an additional affected cow and 2 other seropositive cows; culture of a bulk test of the produced milk was also positive. Subsequently, the entire farm was culled, per state regulations (*5*). Further investigation into the agricultural community identified another affected dairy farm (farm 2); 42 (54.5%) of 77 cows were seropositive. Milk cultures tested positive for *B. abortus* in 4 cows, including 3 that had suffered late abortions; 1 cow also had a positive vaginal culture. All cows on that farm were culled as well. Two domestic dogs residing on farm 2 and 1 dog on farm 1 were also seropositive. Results of serologic testing in 2 nearby dairy farms (housing 79 and 12 cows) were negative, as was testing of bulk milk culture, and no further intervention was pursued in those farms.

Cattle housed in feedlots in this region are sourced through importation from several nonendemic countries and are regulated by the Israeli Ministry of Agriculture and Food Security. Because normal farm turnover meant the cattle present at the time of the investigation in nearby feedlots were not the same as those present during the outbreak, testing cattle in feedlots was considered unnecessary.

Two months after the outbreak was recognized, 1 of the affected dairy farm owners sought care at a local hospital with culture- and serology-confirmed brucellosis. Analysis of the patient’s blood isolate at the NRL confirmed human *B. abortus* infection. Despite ongoing surveillance, no additional *B. abortus* cases were identified.

We further analyzed 6 representative *Brucella* isolates (3 from farm 1, 2 from farm 2, and 1 patient isolate) (Appendix 1 Table 1; Appendix 2 Table). All isolates were confirmed as *B. abortus*. However, the isolates were untypeable, and the specific *B. abortus* biovar could not be determined. Results were confirmed independently at the NRL in Jena, Germany.

**Figure 1:**
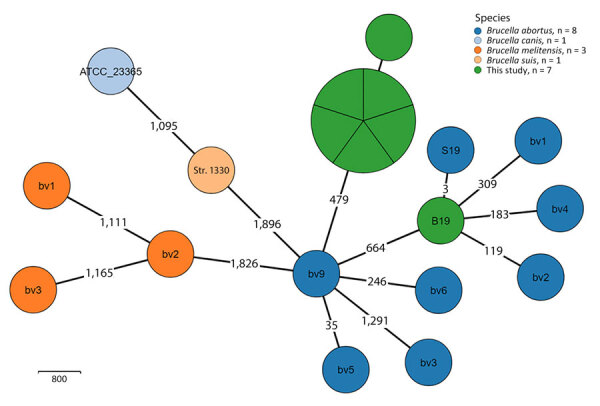
Minimum-spanning tree of 7 study isolates and 13 reference genomes in study of reemergence of *Brucella abortus,* Israel, 2021. Tree shows core-genome multilocus sequence typing analysis of 7 study isolates of *B. abortus* (5 bovine isolates, 1 clinical human isolate, and 1 B19 vaccine strain) and 13 reference *Brucella* genomes (Appendix 1 Table 3), using an ad hoc scheme of 2,424 loci (at 95% genome presence threshold). Node size is proportional to the number of genomes. Numbers denote the number of differing alleles.

**Figure 2:**
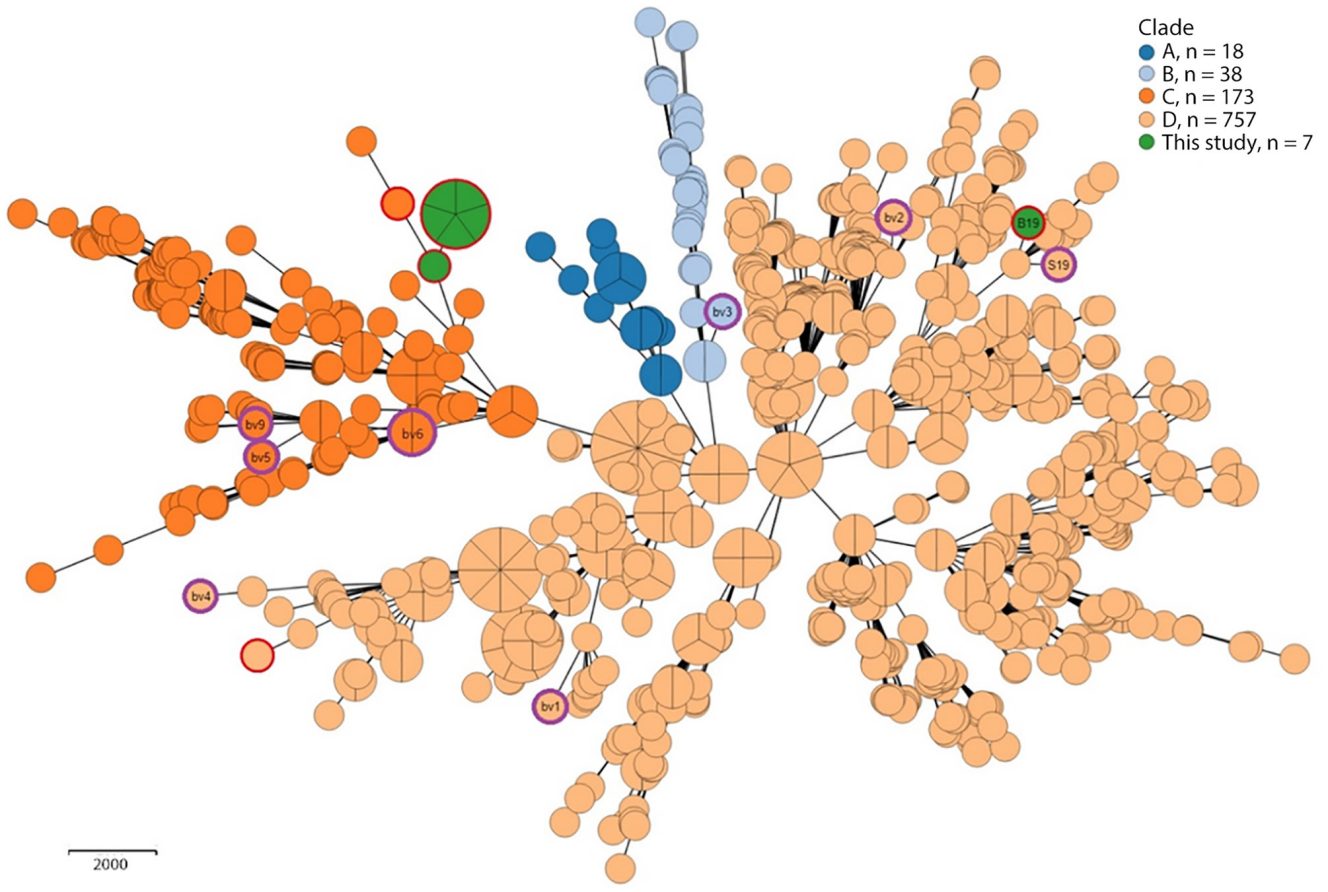
Minimum-spanning tree 7 study isolates and 986 public genomes in study of reemergence of *Brucella abortus,* Israel, 2021. Tree shows core-genome multilocus sequence typing analysis of 7 study isolates of *B. abortus* (5 bovine isolates, 1 clinical human isolate and 1 B19 vaccine strain) and 986 public genomes (Appendix 1 Table 3, using an ad hoc scheme of 2,460 loci (at 95% genome presence threshold). Genomes are color-coded by assigned clade (*6*). Purple outer rings indicate *B. abortus* reference genomes (7 biovars and S19 vaccine strain); red outer rings indicate Isolates from Israel (7 study isolates, 2 public genomes). Node size is proportional to the number of genomes.

To further characterize the outbreak strain, we complemented phenotypic testing with whole-genome sequencing (Appendix 1 Table 2; Appendix 2). The analysis confirmed the taxonomical assignment as *B. abortus*. Phylogenetic analysis using core-genome multilocus sequence typing confirmed all outbreak isolates were clonal (Figure 1) and belonged to *B. abortus*. They did not cluster with any of the known *B. abortus* biovars or the live vaccine strain B19 used in Israel, ruling out the possibility of infection with the vaccine strain. To rule out vaccine contamination with a field strain, the B19 vaccine batches used in the affected farms were cultured, and the recovered isolates were phenotypically confirmed as vaccine strains. The outbreak strain was analyzed with an international collection of *B. abortus* genomes (Appendix 1 Table 3); it belonged to clade C and did not cluster with any publicly available *B. abortus* genomes. The nearest neighbor was an epidemiologically unrelated historical isolate from Israel, showing a notable difference of 93 alleles (Figure 2).

Wild animals inhabiting the surrounding regions (Golan Heights and Galilee) were prospectively sampled to explore the possibility of a wildlife source of infection, including deer, jackals, and wild boar. None of 117 animals tested during 2020–2022 (Appendix 2 Table) were seropositive for brucellosis.

## Conclusions

We describe an outbreak of *B. abortus* occurring in a country considered free of this pathogen for nearly 40 years. The outbreak manifested with cattle abortion and was associated with a high attack rate in 2 affected farms that necessitated the culling of all affected animals, a secondary human case, and 3 infected farm canines. Despite a comprehensive investigation, we could not identify the source of this outbreak. Our genomic analysis did not point to any related strain among publicly available sequences, but this finding does not rule out cryptic cross-border dissemination that might have occurred. Imported beef cattle could be another potential source that cannot be ruled out. Nevertheless, the regulated sourcing of cattle from certified nonendemic countries and the lack of additional animal or human cases in Israel across farms importing from those sources make this scenario less likely. The lack of cases in other farms across Israel makes contaminated animal feed an unlikely explanation. Artificial insemination is also an unlikely source because cattle sperm was sourced locally from certified donors.

The detection of *B. abortus* infection among local dogs is not surprising, given the close contact between canines and farm animals (*7*–*9*). Because the 2 affected farms neither share ownership or farming processes nor exchange animals, free-ranging domestic dogs might have acted as a vehicle of transmission between farms, as previously suggested (*10*). In addition, the affected community is located relatively close to international borders in northern Israel. Although *B. melitensis* is the main species reported in this region (*11*–*14*), the cross-border transmission of *B. abortus* through wildlife or feral dogs remains a possibility.

The outbreak strain was confirmed as *B. abortus* by a range of phenotypic, molecular, and genomic methods as detailed previously. All outbreak isolates exhibited an identical phenotype (Appendix 1 Table 1), but this strain could not be assigned to any known biovars of this species (Appendix 2). Nevertheless, studies of *B. abortus* (and also *B. melitensis*) have demonstrated that isolates belonging to the same biovar do not necessarily cluster phylogenomically (*1*,*3*) (https://www.woah.org/en/what-we-do/standards/codes-and-manuals/terrestrial-manual-online-access). Therefore, although the biovar assignment might have been important traditionally, its epidemiologic relevance might be questionable.

The reemergence of *B. abortus* in a country in which this pathogen has been eliminated for several decades emphasizes the need for continued surveillance, the challenges of tracking reemergence in ecologically complex regions, and the factors underlying the failure of eradication (*15*). This outbreak is especially noteworthy in the epidemiologic context of Israel, where *B. melitensis* is endemic and regularly affects cattle as an accidental host. Although the exact source of this outbreak remains unknown, our investigation exemplifies a possible methodological approach in response to future cases of *B. abortus*. This outbreak highlights the critical need for continuous global surveillance of brucellosis, because the resurgence of zoonotic diseases poses a substantial threat to both animal and human health, as well as to food security worldwide.

Appendix 1 Additional information from study of reemergence of *Brucella abortus,* Israel, 2021

Appendix 2 Additional details regarding study of reemergence of *Brucella abortus,* Israel, 2021
