# Supplementary material for: Reemergence of Brucella abortus, Israel, 2021
Source: Emerg Infect Dis. 2025 Apr;31(4):820–3. doi: 10.3201/eid3104.241003 (PMC11950253; doi:10.3201/eid3104.241003)
Supplement: Appendix 2 — Additional details regarding study of reemergence of Brucella abortus, Israel, 2021 [file 24-1003-Techapp-s2.pdf]

Article DOI: https://doi.org/10.3201/eid3104.241003

EID cannot ensure accessibility for Supplemental Materials supplied by authors. Readers who have difficulty accessing supplementary content should contact the authors for assistance.

Reemergence of *Brucella abortus,* Israel, 2021

Appendix 2

Supplementary Methods

Serologic testing of animals

Bovine blood samples were obtained from the jugular vein or tail and centrifuged at 3,000 rpm for 4 minutes. Sera were tested using in-house serologic assays set up as previously described (*1*) Total antibodies were tested using the complement fixation test (CFT), for which a titer >1:5 was considered positive. In cases of cattle abortion, testing for IgM antibodies was performed as well, using the buffered plate agglutination test (BPAT), for which a titer >1:40 was considered positive.

Microbiological sampling and culture

Bovine sampling for attempted isolation of *Brucella* sp. included bulk milk, udder secretions or vaginal swabs. The collected samples were promptly transported to the National Reference Laboratory (NRL) and processed immediately. Milk samples were left to settle at 4°C overnight. Samples were inoculated by direct culture on solid agar media containing 5% equine serum and supplemented with antibiotics (bacitracin, polymyxin B, nalidixic acid, vancomycin, cycloheximide, and nystatin). The plates were incubated at 37°C±2°C in 5% CO_2.

Phenotypic characterization

Growing isolates were identified at the genus level by conventional microbiological methods (*1*,*2*) and biotyped as previously described based on the following characteristics (Appendix 1 Table 1, https://wwwnc.cdc.gov/EID/article/31/4/24-1003-App1.xlsx): requirement of CO_2 for growth; colonial morphology; growth on dyes (basic fuchsin and thionin); agglutination with monospecific antisera for A and M antigens; H_2S production; urease activity;

oxidase test, inhibition of growth in the presence of penicillin, streptomycin and erythritol; and lysis by the Tbilisi and Iz phages (*1*,*2*).

Molecular characterization

Isolates were grown in pure culture and subjected to DNA extraction using the DNeasy Blood and Tissue kit (QIAGEN, Hilden, Germany) per the manufacturer's protocol. Extracts were subject to PCR assays, including AMOS PCR, as previously described, to confirm the species assignment and differentiate field and vaccine *B. abortus* strains (*3*–*5*).

Whole genome sequencing (WGS)

Sequencing was performed on seven selected isolates representing the two affected farms, different cultured sources (clinical animal and animal milk samples) and human infection (Appendix 1 Table 2). Isolates were grown in pure culture and subjected to DNA extraction using the DNeasy Blood and Tissue kit (QIAGEN, Hilden, Germany) per the manufacturer's protocol. DNA was measured using NanoDrop2000 (ThermoFisher, Waltham, MA, USA), aiming for a 1.8–2.0 A_{260}/A_{280} ratio. DNA libraries for Illumina sequencing were prepared with the Nextera Flex kit (Illumina, San Diego, CA, USA) according to the manufacturer's recommendations, followed by paired-end short-read (SR) 150bp sequencing on an Illumina sequencer (Illumina, San Diego, CA, USA).

Bioinformatics analysis

Short reads from Illumina sequencing underwent quality control (QC, using FastQC v.0.11.5 (https://www.bioinformatics.babraham.ac.uk/projects/fastqc), species identification (using kraken2 (*6*), v.2.0.7-β with the Standard-8 database [from https://benlangmead.github.io/aws-indexes/k2, accessed in February 2023]), trimming (using Trimmomatic (*7*), v.0.39), assembly (using SPADes (*8*), v.3.14.0; and Pilon (*9*), v.1.23), and multilocus sequence typing (MLST) assignment using mlst v.2.23.0 (https://github.com/tseemann/mlst), with pubMLST (*10*) *Brucella spp.* scheme), through the INNUca pipeline v4.2.3, using default parameters (https://github.com/tseemann/mlst). Genome assemblies were checked for quality using CheckM2 (*11*) (v.1.0.1; with the default database) and species identification was confirmed as *Brucella abortus* using gambit (*12*) (v.1.0.0; with the default gambit-refseq-curated-1.0 database).

For phylogenetic comparison to other *Brucella* species, 13 reference *Brucella* genome assemblies, including the three *B. melitensis* biovars (bv1 [GCF_000007125.1], bv2 [GCF_000740395.1] and bv3 [GCF_000740355.1]), the seven *B. abortus* biovars (bv1 [GCA_000739315.1], bv2 [GCA_000740375.1], bv3 [GCA_000157715.1], bv4 [GCA_000742275.1], bv5 [GCA_000163115.1], bv6 [GCA_000740215.1], and bv9 [GCA_000740195.1]), the vaccine strain *B. abortus* S19 (GCA_000018725.1), *B. suis* strain 1330 (GCF_000007505.1) and *B. canis* ATCC_23365 (GCF_000018525.1) were downloaded from NCBI Genbank or Refseq using the tool datasets v.16.9.0 (https://github.com/ncbi/datasets); with the parameters "accession {NCBI accession}–include genome." For comparison to the global *B. abortus* genome collection, an additional 986 publicly available genome assemblies (from [PMC10716283] (*13*) Appendix 1 Table 3) were downloaded from the AllTheBacteria dataset v.0.2 (https://ftp.ebi.ac.uk/pub/databases/AllTheBacteria/Releases/0.2/) and NCBI Genbank or Refseq using the tool datasets (as mentioned above).

Ad hoc core-genome multilocus sequence typing (cgMLST) analysis of the genome assemblies of the outbreak isolates from this study was conducted in two contexts: 1) in comparison to reference *Brucella* species and biovars, using the 13 reference *Brucella* genomes (mentioned above) and the study isolates (n = 7) to generate an ad hoc cgMLST scheme consisting of 2,424 loci (Figure 1); 2) in comparison to global *B. abortus* genome collection, using the 986 *B. abortus* genomes listed in [PMC10716283] and the study isolates (n = 7) to generate an ad hoc cgMLST scheme consisting of 2,460 loci (Figure 2); Both ad hoc schemas were generated using chewBBACA (*14*) (v.3.2.0) [with a Prodigal (*15*) (v.2.6.3) training file for the reference genome GCA_000739315.1 and including only loci with at least 95% genome presence]. Minimum spanning trees were generated (with MSTreeV2) and visualized from the ad hoc cgMLST scheme using GrapeTree (*16*) (v.1.5.0).

WGS data were deposited to ENA under the bioproject ID: PRJEB77195.

Ethical Approval

This work was approved by the Ethics Committee of the Hadassah Medical Center (approval number HMO-23–0523).

Supplementary Results

The outbreak strain could not be typed using the above phenotypic tests. As compared to biovars that are typically positive for agglutination with *abortus* monospecific serum (as was the outbreak strain), its positive urease activity differentiated it from biovar 1, growth in the presence of fuchsin differentiated it from biovar 2, inhibited growth in the presence of thionin differentiated it from biovar 3, and CO_2 growth requirement, growth in the presence of thionin and H_2S production differentiated it from biovar 6.

Appendix 2 Table. Wildlife tested in the outbreak region 2020–2022

Animal species	Year	No. tested	No. positive
Wild Boar	2020	7	0
	2021	0	0
	2022	7	0
Deer	2020	35	0
	2021	5	0
	2022	34	0
Wild Goat	2020	6	0
	2021	0	0
	2022	0	0
Jackal	2020	14	0
	2021	0	0
	2022	0	0
Spotted Deer	2020	1	0
	2021	0	0
	2022	0	0
Gazelle	2020	0	0
	2021	0	0
	2022	8	0

References

1. Alton GG, Jones LM, Angus RD, Verger JM. Techniques for the brucellosis laboratory. Paris: INRA Publications; 1988.

2. World Organisation for Animal Health. Terrestrial manual online access [cited 2024 Jul 6]. https://www.woah.org/en/what-we-do/standards/codes-and-manuals/terrestrial-manual-online-access/

3. Bardenstein S, Mandelboim M, Ficht TA, Baum M, Banai M. Identification of the *Brucella melitensis* vaccine strain Rev.1 in animals and humans in Israel by PCR analysis of the PstI site polymorphism of its omp2 gene. J Clin Microbiol. 2002;40:1475–80. PubMed https://doi.org/10.1128/JCM.40.2.1475-1480.2002

4. Le Flèche P, Jacques I, Grayon M, Al Dahouk S, Bouchon P, Denoeud F, et al. Evaluation and selection of tandem repeat loci for a Brucella MLVA typing assay. BMC Microbiol. 2006;6:9. PubMed https://doi.org/10.1186/1471-2180-6-9

5. Bricker BJ, Halling SM. Enhancement of the Brucella AMOS PCR assay for differentiation of Brucella abortus vaccine strains S19 and RB51. J Clin Microbiol. 1995;33:1640–2. PubMed https://doi.org/10.1128/jcm.33.6.1640-1642.1995

6. Wood DE, Lu J, Langmead B. Improved metagenomic analysis with Kraken 2. Genome Biol. 2019;20:257. PubMed https://doi.org/10.1186/s13059-019-1891-0

7. Bolger AM, Lohse M, Usadel B. Trimmomatic: a flexible trimmer for Illumina sequence data. Bioinformatics. 2014;30:2114–20. PubMed https://doi.org/10.1093/bioinformatics/btu170

8. Bankevich A, Nurk S, Antipov D, Gurevich AA, Dvorkin M, Kulikov AS, et al. SPAdes: a new genome assembly algorithm and its applications to single-cell sequencing. 2012;19:455–77. **PMID 22506599**

9. Walker BJ, Abeel T, Shea T, Priest M, Abouelliel A, Sakthikumar S, et al. Pilon: an integrated tool for comprehensive microbial variant detection and genome assembly improvement. PLoS One. 2014;9:e112963. PubMed https://doi.org/10.1371/journal.pone.0112963

10. Jolley KA, Bray JE, Maiden MCJ. Open-access bacterial population genomics: BIGSdb software, the PubMLST.org website and their applications. Wellcome Open Research 2018 3:124. 2018 Sep 24;3:124. https://doi.org/10.12688/wellcomeopenres.14826.1

11. Chklovski A, Parks DH, Woodcroft BJ, Tyson GW. CheckM2: a rapid, scalable and accurate tool for assessing microbial genome quality using machine learning. Nat Methods. 2023;20:1203–12. PubMed https://doi.org/10.1038/s41592-023-01940-w

12. Lumpe J, Gumbleton L, Gorzalski A, Libuit K, Varghese V, Lloyd T, et al. GAMBIT (Genomic Approximation Method for Bacterial Identification and Tracking): a methodology to rapidly leverage whole genome sequencing of bacterial isolates for clinical identification. PLoS One. 2023;18:e0277575. PubMed https://doi.org/10.1371/journal.pone.0277575

13. Janke NR, Williamson CHD, Drees KP, Suárez-Esquivel M, Allen AR, Ladner JT, et al. Global phylogenomic diversity of *Brucella abortus*: spread of a dominant lineage. Front Microbiol. 2023;14:1287046 PubMed https://doi.org/10.3389/fmicb.2023.1287046

14. Silva M, Machado MP, Silva DN, Rossi M, Moran-Gilad J, Santos S, et al. chewBBACA: A complete suite for gene-by-gene schema creation and strain identification. Microb Genom. 2018;4:e000166. PubMed https://doi.org/10.1099/mgen.0.000166

15. Hyatt D, Chen GL, Locascio PF, Land ML, Larimer FW, Hauser LJ. Prodigal: prokaryotic gene recognition and translation initiation site identification. BMC Bioinformatics. 2010;11:119. PubMed https://doi.org/10.1186/1471-2105-11-119

16. Zhou Z, Alikhan NF, Sergeant MJ, Luhmann N, Vaz C, Francisco AP, et al. GrapeTree: visualization of core genomic relationships among 100,000 bacterial pathogens. Genome Res. 2018;28:1395–404. PubMed https://doi.org/10.1101/gr.232397.117
